# Exudate Unidirectional Pump to Promote Glucose Catabolism Triggering Fenton‐Like Reaction for Chronic Diabetic Wounds Therapy

**DOI:** 10.1002/advs.202404652

**Published:** 2024-08-09

**Authors:** Yaxian Liang, Wenjie Wang, Kailong Qi, Yige Wei, Weifeng Zhao, Huixu Xie, Changsheng Zhao

**Affiliations:** ^1^ State Key Laboratory of Oral Diseases National Center for Stomatology National Clinical Research Center for Oral Diseases Department of Head and Neck Oncology West China Hospital of Stomatology Sichuan University Chengdu 610041 China; ^2^ College of Polymer Science and Engineering State Key Laboratory of Polymer Materials Engineering Sichuan University Chengdu 610054 China

**Keywords:** antibacterial, diabetic infected wound, exudate pump, Fenton‐like reaction, Janus membrane, localized glucose decomposition

## Abstract

The massive accumulation of exudate containing high concentrations of glucose causes wound infection and triggers the release of inflammatory factors, which in turn delays the closure of diabetic wounds. In this study, a Janus membrane is constructed by combining glucose oxidase (GOx) and copper ions (Cu^2+^) for the treatment of diabetic wounds, which is named as Janus@GOx/Cu^2+^. It consists of hydrophobic, transitional, and superhydrophilic layers in a three‐layer structure with gradient hydrophilicity for self‐pumping properties. The Janus@GOx/Cu^2+^ membrane triggers a series of cascading reactions while pumping out diabetic wound exudates. First, glucose oxidase loaded onto the hydrophilic layer of the Janus@GOx/Cu^2+^ membrane decomposes glucose into hydrogen peroxide (H_2_O_2_) and glucuronic acid, reducing the local glucose level. The generated glucuronic acid neutralizes the local alkaline environment of chronic wounds. Simultaneously, the H_2_O_2_ interacts with the Cu^2+^ contained in the hydrophobic layers of the Janus@GOx/Cu^2+^ membrane via a Fenton‐like reaction, generating hydroxyl radicals with excellent bactericidal properties. Cu^2+^ promotes angiogenesis and wound healing in diabetic wounds. Under the action of multiple responses, the Janus@GOx/Cu^2+^ membrane promotes wound healing in diabetic infections.

## Graphical abstract

A three‐layer Janus electrostatically spun membrane loaded with glucose oxidase and copper ions is designed, which shows great potential in diabetic wound healing. It enables to pump out of wound exudate unidirectionally while triggering a series of cascade reactions to decompose localized glucose, generate ·OH with strong oxidative properties, and exert excellent antimicrobial function.

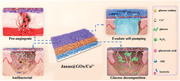

## 1 Introduction

Diabetes is the fastest‐growing global health emergency of the 21^st^ century.^[^ ^1^ ^]^ Patients with diabetes often suffer from chronic injures, such as diabetic foot and diabetic ulcers. The delayed healing of diabetic wounds can be attributed to several factors, including high local glucose concentrations, vascular disease, neuropathy, infections, immune system defects, disturbances in growth factor activity, cellular dysfunction, and poor oxygenation.^[^ ^2^ ^]^

After skin injury, changes in the local microenvironment and inflammatory responses cause vasodilatation and increased permeability, resulting in wound exudate generation. Excessive exudates prolong the inflammatory period, interfere with the action of growth factors, and delay wound healing.^[^ ^3^ ^]^ The overflowing exudate also leads to peri‐wound maceration, which means that the peri‐wound skin is overhydrated, damaging its integrity and losing protection against infection.^[^ ^4^ ^]^ In infected diabetic wounds, a constant exudate with a high level of glucose supplies nutrients for bacterial growth and biofilm formation, reducing the penetration and bactericidal effects of conventional antibiotics.^[^ ^5^ ^]^ On the one hand, the metabolism of microorganisms attached to the wound leads to insufficient oxygenation and nutrients in the wound tissue.^[^ ^6^ ^]^ Conversely, metabolites such as lactate and reactive oxygen species, along with excessive advanced glycosylation end products, trigger an intense immune response from the host, leading to the release of inflammatory factors, causing oxidative damage, and inducing changes in the extracellular matrix. Additionally, the immune response inhibits the proliferation of fibroblasts, endothelial cells, and keratin‐forming cells.^[^ ^7^ ^]^ Diabetes mellitus is also associated with microvascular disease. In wounds, reduced peripheral blood flow and local neovascularization due to microangiopathy reduce the supply of oxygen and nutrients to the wound bed, which are significant obstacles to diabetic wound healing.^[^ ^8^ ^]^ For these reasons, diabetic wounds are more complicated to heal than normal wounds. Therefore, advanced wound dressings targeting diabetic wounds are in high clinical demand.

Traditional wound dressings such as gauze and bandages work by hemostasis and isolation from the external environment, rather than maintaining local moistening of the wound. Persistent exuding wounds tend to adhere with the dressings and may cause secondary damage.^[^ ^9^ ^]^ Modern wound dressings with better biocompatibility and moisturization could relieve wound pain and maintain local humidity while moving out excess wound exudate.^[^ ^6^, ^10^ ^]^ Janus membranes with self‐pumping functions are promising emerging biomaterials. The self‐pumping function is usually achieved by constructing an asymmetric membrane with opposite moisture wettability properties on the front and back sides, which unidirectionally drains excess biofluid away from the wounds.^[^ ^11^ ^]^ These Janus membranes pump out exudate unidirectionally, reducing local exudate impregnation of the peri‐wound. To promote the healing of diabetic wounds, functional Janus membranes are loaded with antibacterial agents, such as ciprofloxacin, amoxicillin, and curcumin, to achieve antimicrobial effects, as well as insulin to achieve local hypoglycemia. However, the limited drug loading and uneven drug release of conventional self‐pumping dressings make it difficult to achieve sustained antimicrobial effects. Additionally, topical use of antibiotics may lead to the emergence of multidrug‐resistant bacteria. Moreover, insulin takes a longer time to reach the target cells through the body's circulation after topical application. Therefore, new local antimicrobial and microenvironmental modulation strategies must be developed to treat diabetic wounds.

**Figure 1:**
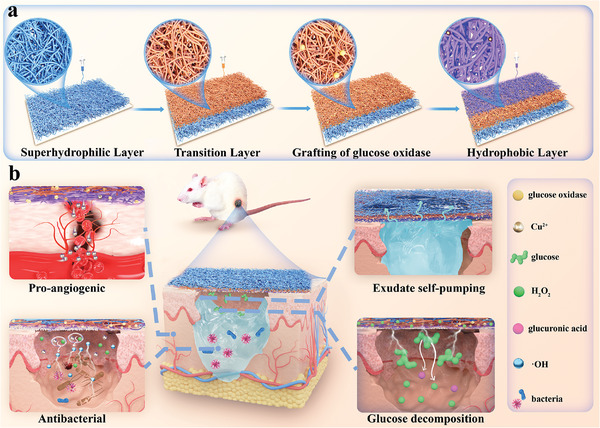
Preparation and application of self‐pumping Janus@GOx/Cu^2+^ membrane. a) Fabrication of Janus@GOx/Cu^2+^ membrane loaded with GOx and Cu^2+^. b) Janus@GOx/Cu^2+^ promotes diabetic infected wound healing by exudate drainage, local glucose reduction, antimicrobial effect and angiogenesis promotion.

To solve these problems, a Janus membrane was constructed that combines glucose oxidase (GOx) and copper ions (Cu^2+^), known as Janus@GOx/Cu^2+^ (**Figure** **1**a). Polyacrylonitrile (PAN) fibers were used as superhydrophilic layers after the alkali treatment. Alkali treatment was aimed at enhancing the hydrophilicity of the PAN fibers. Polyurethane (PU) fibers containing copper sulfate pentahydrate were used as hydrophobic layers to introduce Cu^2+^ into the system. To improve the connection between the superhydrophilic and hydrophobic layers, an intermediate transition layer was prepared by co‐electrospinning PAN and PU. The insertion of the intermediate transition layer constructed a progressive wettability between the three layers, which is able to guide the continuous transport of water through the fiber membrane. It prevented the rewetting of the inner layer on the one hand, and avoided the inability of water transport due to the over‐thickness of the hydrophobic layer on the other hand.^[^ ^12^ ^]^ GOx was grafted onto PAN fibers on the superhydrophilic and transition layers of Janus@GOx/Cu^2+^ membranes (Figure S1, Supporting Information). The Janus@GOx/Cu^2+^ membrane triggers a cascade reaction while pumping out localized exudates (Figure 1b). Specifically, the Janus@GOx/Cu^2+^ membrane decreased the glucose concentration and reduced tissue damage from excessive glycosylation by decomposing glucose into hydrogen peroxide (H_2_O_2_) and glucuronic acid via GOx. The generated glucuronic acid neutralizes the local alkaline environment of chronic wounds.^[^ ^13^ ^]^ Subsequently, the introduced Cu^2+^ performs a Fenton‐like reaction with the generated H_2_O_2_ to generate hydroxyl radicals (·OH) with high chemical reactivity and short half‐life. The ·OH provides robust and broad‐spectrum bactericidal activity while avoiding potential damage to deeper tissues.^[^ ^13^, ^14^ ^]^ On the other hand, the Janus@GOx/Cu^2+^ membrane offers an appropriate level of Cu^2+^ to the wound sites, which promotes angiogenesis and migration of keratinocytes and fibroblasts by promoting the expression of vascular endothelial growth factor (VEGF) and hypoxia‐inducible factor‐1α (HIF‐1α), as well as keratin and collagen.^[^ ^15^ ^]^ In summary, the Janus@GOx/Cu^2+^ membrane provided new insights into diabetic wound management.

## 2 Results

### 2.1 Preparation and Characterization of the Nanofiber Membranes

First, the nanofiber membranes were prepared by electrospinning. In the Janus@GOx/Cu^2+^ membrane, GOx was loaded onto the superhydrophilic and transition layers by grafting, and Cu^2+^ was loaded onto the hydrophobic layer by blending. The Cu^2+^ concentration in the Janus@GOx/Cu^2+^ membrane was optimized by evaluating the toxicity of different Cu^2+^ concentrations in L929 cells. According to the CCK‐8 results shown in Figure S2 (Supporting Information), the Cu^2+^ concentration in the hydrophobic layer spinning solution was determined to be 5 mmol L^−1^, because the Janus@GOx/Cu^2+^ membrane exhibited good cytocompatibility at this concentration. We detected the release of Cu^2+^ by inductively coupled plasma optical emission spectrometry (ICP‐OES). The concentrations of Cu^2+^ released from 1 cm^3^ Janus@GOx/Cu^2+^ membranes were 1.29 ± 0.22  and 1.91 ± 0.17 µmol L^−1^ after 12 and 24 h, respectively, which were lower than the cytotoxic and bactericidal concentrations.^[^ ^16^ ^]^ It is further shown that the release of Cu^2+^ is traced, with good cytocompatibility, and Cu^2+^ is majorly loaded on the fiber membrane to perform its functions.

**Figure 2:**
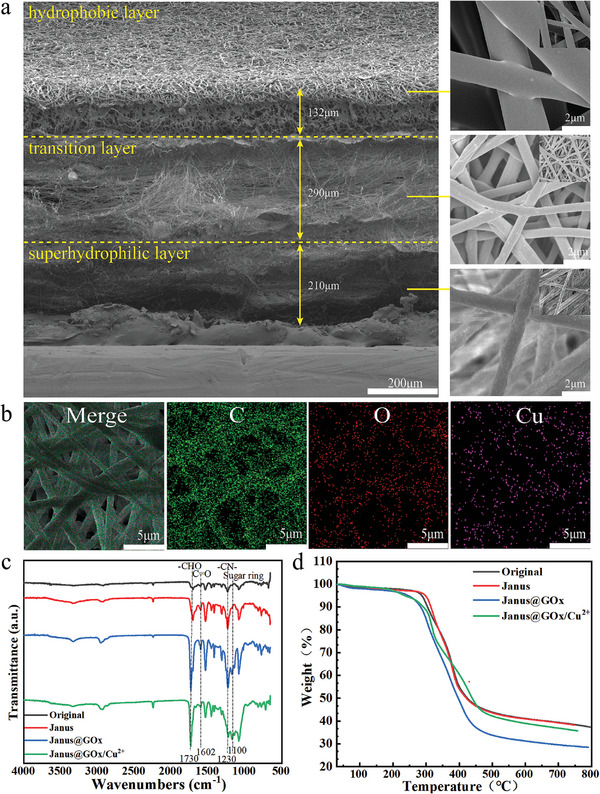
a) The SEM image of the cross‐section of the Janus@GOx/Cu^2+^ membrane and the more microscopic images of hydrophobic layer, superhydrophilic layer and transition layer. Scale bar b) EDS mappings of C, O, and Cu in the hydrophobic layer of Janus@GOx/Cu^2+^ membranes. c) FTIR and d) TGA of the membranes. Scale bars are marked in the pictures.

As shown in the scanning electron microscopy (SEM) images (**Figure** **2**a), the Janus@GOx/Cu^2+^ membrane exhibits a uniform and slender fiber morphology. The cross‐sectional SEM image clearly shows the three‐layered structure of the Janus@GOx/Cu^2+^ membrane, including a hydrophobic layer (thinnest), a transition layer (thickest), and a superhydrophilic layer. After the hydrophilic PAN fibers were hydrolyzed using the alkaline solution, the surface roughness became pronounced, which improved the hydrophilicity of Janus@GOx/Cu^2+^, and resulted in the formation of a superhydrophilic layer. The roughness of the transition‐layer fibers was lower than that of the superhydrophilic layer fibers after alkaline treatment due to the addition of PU. In the hydrophobic layer, the fiber surface remained smooth because the addition of copper sulfate pentahydrate did not alter the morphology. Energy‐dispersive spectrometry (EDS) mapping (Figure 2b) confirmed that Cu was homogeneously distributed on the membrane. As shown in Figure 2c, the changes in the chemical structure of the membranes were analyzed using Fourier‐transform infrared (FTIR) spectroscopy. The original membrane is referred to as the trilaminar membrane, which was not treated with alkali and was not loaded with GOx or Cu^2+^. The Janus membrane refers to a trilaminar membrane treated with an alkali but not loaded with GOx or Cu^2+^. The Janus@GOx membrane is referred to as a trilaminar membrane treated with alkali and loaded only with GOx. First, PAN was reacted with an alkali to form polyacrylamide. During this reaction, the nitrile group (─C≡N) was converted to an amide group (─CONH_2_). This conversion resulted in the peaks caused by the stretching vibration of ─C─NH_2_ at 1230 cm^−1^ and the stretching vibration of C═O at 1602 cm^−1^ becoming more potent in the FTIR spectrum of Janus, Janus@GOx and Janus@GOx/Cu^2+^ membranes compared to the original membrane. Next, the amino group of polyacrylamide underwent a crosslinking reaction with the carbonyl group of glutaraldehyde, incorporating the aldehyde group into the polymer structure. As a result, the characteristic absorption peaks of ─CHO at 1730 cm^−1^ in Janus@GOx and Janus@GOx/Cu^2+^ became stronger. Subsequently, the aldehyde group from the glutaraldehyde underwent a condensation reaction with the amino group of GOx. This reaction allowed the grafting of GOx onto the PAN fibers. Grafting of GOx was indicated by the appearance of a characteristic peak of the sugar ring at 1100 cm^−1^ in the FTIR spectra of the Janus@GOx and Janus@GOx/Cu^2+^ membranes. The thermal stabilities of the nanofiber membranes were investigated using thermogravimetric analysis (TGA). As shown in Figure 2d, the fiber membranes had good thermal stability at 250 °C and were applicable under normal conditions. Compared with the Original membrane, weight loss started at a higher temperature in the Janus membrane, which was related to a reduction in volatile components after alkali treatment. However, the weight‐stability temperatures for both groups remained relatively constant, indicating that the structure and composition of the materials did not undergo significant changes. Janus@GOx showed earlier weight loss and a significantly higher percentage of mass loss than the Original and Janus membranes. This is attributed to the grafting of GOx. GOx undergoes thermal decomposition at temperatures of 200–300 °C, resulting in significant weight loss of Janus@GOx.^[^ ^17^ ^]^ The substantial weight loss of Janus@GOx/Cu^2+^ was observed at 250 °C due to the thermal decomposition of the organic component GOx. However, the addition of the inorganic component copper sulfate pentahydrate helped reduced the percentage of weight loss. Thus, the Janus@GOx/Cu^2+^ exhibited a lower weight loss ratio than Janus@GOx.

### 2.2 Self‐Pumping Behavior and Mechanism of Janus Membranes

**Figure 3:**
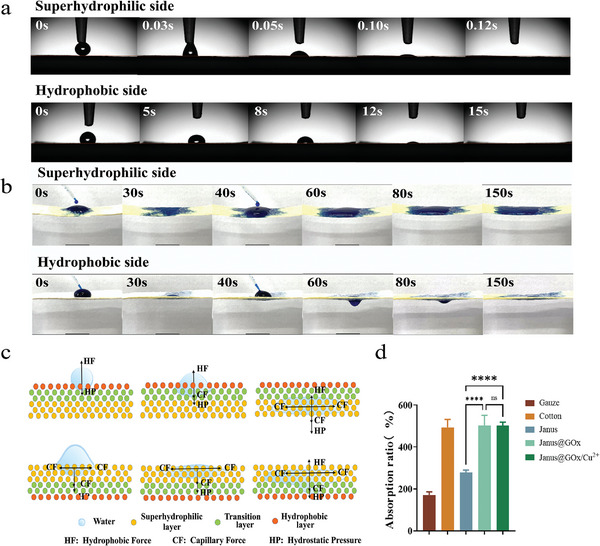
a) WCA tests on superhydrophilic and hydrophobic sides of the Janus@GOx/Cu^2+^ membrane. b) Lateral view of the water transport process in the Janus@GOx/Cu^2+^ membrane. c) Schematic illustration of the directional water transport mechanism. d) The liquid absorption ratios of the membranes. Data were expressed as mean ± SD. *n* ≥ 3, *p < 0.05; **p < 0.01; ***p < 0.001, ****p < 0.0001, and ns for no significant differences.

A three‐layer membrane structure with progressive wetting properties exhibits moisture‐directed transport properties.^[^ ^12b^ ^]^ In this study, a three‐layer Janus electrospun membrane with a gradient wettability was designed to achieve unidirectional permeability. As shown in **Figure** **3**a, the water contact angles on the superhydrophilic and hydrophobic sides of Janus@GOx/Cu^2+^ were tested. When the droplets reached the superhydrophilic side, they were rapidly absorbed. However, the hydrophobic side of Janus@GOx/Cu^2+^ with gradual wettability triggered slow absorption of the droplet, which was completely absorbed within 15 s. An ink‐diffusion experiment was performed to test the directional liquid transport characteristics of Janus@GOx/Cu^2+^ (Figure 3b). When 200 µL of blue ink was dropped on the superhydrophilic side, it was diffused rapidly but blocked by the hydrophobic side, failing to penetrate the hydrophobic layer. After 40 s, another 200 µL of ink was dropped on the superhydrophilic side. All the 400 µL of ink did not penetrate the hydrophobic layer after 150 s, demonstrating the excellent moisture resistance of the inner layer of the Janus@GOx/Cu^2+^. This property allows it to hold more moisture and effectively prevent the penetration of external moisture. When the ink was dropped onto the hydrophobic side, it gradually penetrated the transition layer and diffused into the superhydrophilic layer. After adding another 200 µL of ink, the droplet first exuded from the superhydrophilic side due to gravity. The ink droplet was then entirely absorbed by the Janus@GOx/Cu^2+^ membrane, and the hydrophobic side dried again. Ink reverse osmosis experiments were performed on both the superhydrophilic and hydrophobic sides of Janus@GOx/Cu^2+^. As demonstrated in Movies S1 and S2 (Supporting Information), the ink was injected from the bottom of the Janus@GOx/Cu^2+^ membrane. When injected from the superhydrophilic side, the ink spreads rapidly across the Janus@GOx/Cu^2+^ membrane. In contrast, when injected from the hydrophobic side, the ink was gradually pumped into the transfer layer and completely diffused. We tested the drying of the membrane with a strip of filter paper after dropping ink on both sides of the Janus@GOx/Cu^2+^ membrane, respectively. The filter paper strip was moist after contact with the superhydrophilic side and dry after contact with the hydrophobic side. The superhydrophilic side was stained blue by the diffused ink (Figure S3a, Supporting Information). In contrast, the hydrophobic layer remained dry and clean because of the complete penetration of droplets into the transition layer and superhydrophilic layers (Figure S3b, Supporting Information). The liquid breakthrough pressures of the different monolayer membranes and the Janus@GOx/Cu^2+^ membranes were also measured. As shown in Figure S4 (Supporting Information), compared with the PU monolayer membrane, the breakthrough pressures of the PU/PAN, PAN, and PAN@GOx monolayer membranes decreased, indicating that the addition of PAN and GOx increased the hydrophilicity. For the Janus@GOx/Cu^2+^ membrane, the liquid breakthrough pressure of the hydrophobic side (16.58 cm H_2_O) was much higher than that of the superhydrophilic side (5.84 cm H_2_O). Therefore, the liquid could be pumped unidirectionally from the hydrophobic side to the hydrophilic side.

The liquid transfer principle of the Janus@GOx/Cu^2+^ membrane is summarized by analyzing the different forces exerted on the droplets (Figure 3c). When dripped from the hydrophobic side, a small amount of liquid could not penetrate the Janus@GOx/Cu^2+^ membrane because of the hydrophobic forces (HF) of the fiber on the liquid. However, as the volume of the liquid increases, the hydrostatic pressure (HP) of the liquid exceeds that of the HF, thus the liquid penetrates the hydrophobic layer. Subsequently, the liquid diffused into the transition and superhydrophilic layers under the effect of capillary forces (CF) generated by the pores between the fibers. When dripped from the superhydrophilic side, the liquid spread rapidly in the superhydrophilic and transition layers under the effect of HP and CF. The liquid did not break through the hydrophobic layer because of the HF provided by the hydrophobic layer fibers.

The liquid absorption ratio of the Janus@GOx/Cu^2+^ membranes was evaluated in phosphate‐buffered saline (PBS). As shown in Figure 3d, the absorption ratios of the gauze, cotton, and Janus@GOx/Cu^2+^ membranes are 171%, 493%, and 502%, respectively. The Janus@GOx/Cu^2+^ membrane demonstrated better liquid absorption capability than the gauze. Although the liquid absorption performance of the Janus@GOx/Cu^2+^ membrane was comparable to that of cotton, the Janus@GOx/Cu^2+^ membrane pumped liquids unidirectionally, thereby preventing the accumulation of wound exudate. Additionally, the increase in the absorption ratio of Janus@GOx/Cu^2+^ compared to that of Janus was due to the hydrolysis of the polyacrylonitrile components by the alkali treatment, which further enhanced the hydrophilicity of the fibers.

### 2.3 GOx Activity and Fenton‐Like Activity

The suitable pH range for GOx to catalyze the glucose catabolism reaction is 4–6, and an alkaline environment significantly impacts the activity of GOx.^[^ ^18^ ^]^ Additionally, Cu^2+^ has been proven to exhibit excellent Fenton‐like catalytic activity in the pH range of 3–7.^[^ ^19^ ^]^ The pH of the body is typically maintained at 7.35–7.45, but localized exudation from chronic injury wounds can alter the pH of the wound to 7–9.^[^ ^20^ ^]^ Accordingly, to mimic the chronic diabetic wound environment, GOx and Fenton‐like activities were measured at an initial pH of 8.5 and a glucose concentration of 2.5 g L^−1^.

**Figure 4:**
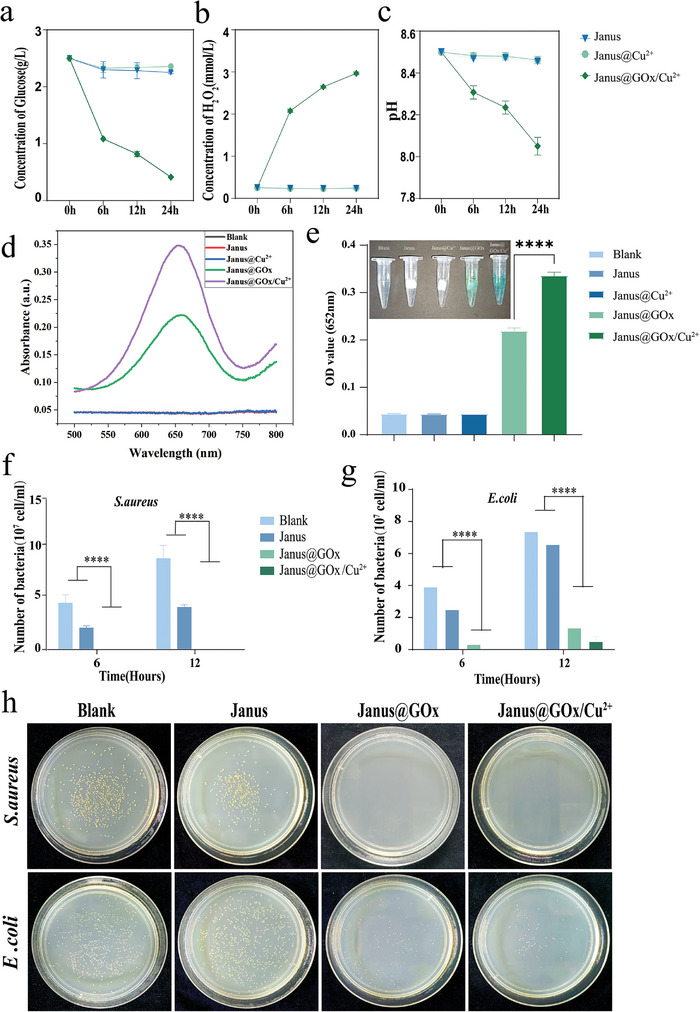
Changes of a) glucose concentration, b) H_2_O_2_ concentration, and c) pH. d) Absorption spectra of TMB chromogenic reaction at 500–800 nm. e) Absorbance values of TMB chromogenic reaction at 652 nm. Statistics of f) *S. aureus* and g) *E. coli* plate counting results. h) Plate counting pictures of *S. aureus* and *E. coli* after 12 h. Data were expressed as mean ± SD. *n* ≥ 3, *p < 0.05; **p < 0.01; ***p < 0.001, ****p < 0.0001, and ns for no significant differences.

**Figure** **4**a–c exhibits the GOx activity of the Janus@GOx/Cu^2+^ membrane. As shown in Figure 4a, GOx played a significant role within the first 6 h, with the glucose concentration decreasing by more than half to about 1 g L^−1^ in both pH environments. At the same time, the concentration of H_2_O_2_ in the solution rose to 2 mmol L^−1^ (Figure 4b), and the pH decreased to about 8.3 due to the production of glucuronic acid (Figure 4c). As the pH decreased, GOx further catalyzed the breakdown of glucose, facilitating product generation and environmental changes. Meanwhile, the Janus and Janus@Cu^2+^ membranes could regulate the wound glucose concentration, H_2_O_2_ concentration, and pH by catalyzing cascade reactions. The GOx on Janus@GOx/Cu^2+^ also exhibited excellent glucose catabolism in an acidic environment at pH = 5.5 (Figure S5a–c, Supporting Information), indicating that this material can also be used for microenvironmental regulation of acute wounds. In addition, we performed an experiment on pH regulation by diffusion of glucuronic acid. As shown in Figure S6 (Supporting Information), the pH of the glucose solution gradually decreased after connecting only with the hydrophobic layer of Janus@GOx/Cu^2+^. This indicated that the glucuronic acid produced in the transfer layer and superhydrophilic layer could diffuse into the solution through the hydrophobic layer to regulate the pH value of the solution. The catalytic activity of the Janus membrane system remained largely unaffected by the alkaline environment, likely due to a potential 90% reduction in the impact of the alkaline environment on glucose oxidase activity in the presence of its substrate, glucose.^[^ ^18^, ^21^ ^]^ Moreover, the structural changes in glucose oxidase caused by the alkaline environment are reversible.^[^ ^22^ ^]^ The activity of glucose oxidase gradually increased as the production of glucuronic acid lowered the solution pH.

Next, the Fenton‐like activity of Janus@GOx/Cu^2+^ was verified by the 3, 3′,5,5′‐tetramethylbenzidine (TMB) colorimetric method. The colorless TMB is oxidized to blue by ·hydroxyl radicals (·OH), along with a characteristic absorption peak at 652 nm. As shown in Figure 4d,e, the TMB solutions co‐cultured with the Janus@GOx and Janus@GOx/Cu^2+^ membranes appeared blue after 24 h. The Janus@GOx/Cu^2+^ group exhibited a higher absorbance at 652 nm than the Janus@GOx group. These results indicated that H_2_O_2_ was generated in the solutions of Janus@GOx and Janus@GOx/Cu^2+^ group, and the ·OH generated from H_2_O_2_ decomposition were chromogenic with TMB. The solution turned blue in the Janus@GOx group probably because of the spontaneous decomposition of H_2_O_2_ to produce ·OH.^[^ ^23^ ^]^ At the same time, the Cu^2+^ on the Janus@GOx/Cu^2+^ reacted with H_2_O_2_ in a Fenton‐like reaction to accelerate the generation of ·OH, making the chromogenic reaction even more significant. As shown in Figure S5d,e (Supporting Information), the Fenton‐like reaction initiated by the Janus@GOx/Cu^2+^ membrane also occurred in an acidic environment at pH 5.5. These results demonstrated the Fenton‐like activity of Janus@GOx/Cu^2+^.

### 2.4 Antibacterial Activity

·OH is an effective agent due to its outstanding antimicrobial properties. The in vitro antibacterial ability of the fiber membranes was next verified. *Staphylococcus aureus* is a common source of infection that secretes multiple toxic factors and shows strong resistance to antibiotics. *Escherichia coli*, on the other hand, is not only a member of the typical mammalian intestinal flora but also a common pathogenic bacterium. These two types of bacteria are Gram‐negative and Gram‐positive. As shown in Figure S7a (Supporting Information), Janus@GOx and Janus@GOx/Cu^2+^ exhibited good bactericidal activity against *S. aureus* after 6 and 12 h, with the absorbance of the bacterial solution similar to that of the sterile medium. Furthermore, the colony counts at 6 h (Figure S8, Supporting Information), and 12 h (Figure 4h) suggested that Janus@GOx/Cu^2+^ showed excellent resistance to *S. aureus*, with the number of colonies on the Petri dishes significantly reduced compared to the other groups. Quantitative statistics showed that it achieved 100% killing ability for *S. aureus* at 6 and 12 h (Figure 4f). Similarly, Janus@GOx/Cu^2+^ showed excellent antibacterial effects against *E. coli* after 6 and 12 h (Figure 4g and Figure S7b, Supporting Information), achieving more than 90% bactericidal activity. The morphology of the bacteria was also observed by SEM. As shown in Figure S9 (Supporting Information), the outer membrane structures of *S. aureus* and *E.coli* were significantly damaged in the Janus@GOx group and JanusGOx/Cu^2+^ group. They were most visibly damaged by JanusGOx/Cu^2+^treatment and showed significant dehydration, shrinkage and breakage of outer membrane.

### 2.5 In Vitro Biocompatibility and Angiogenic Evaluation

**Figure 5:**
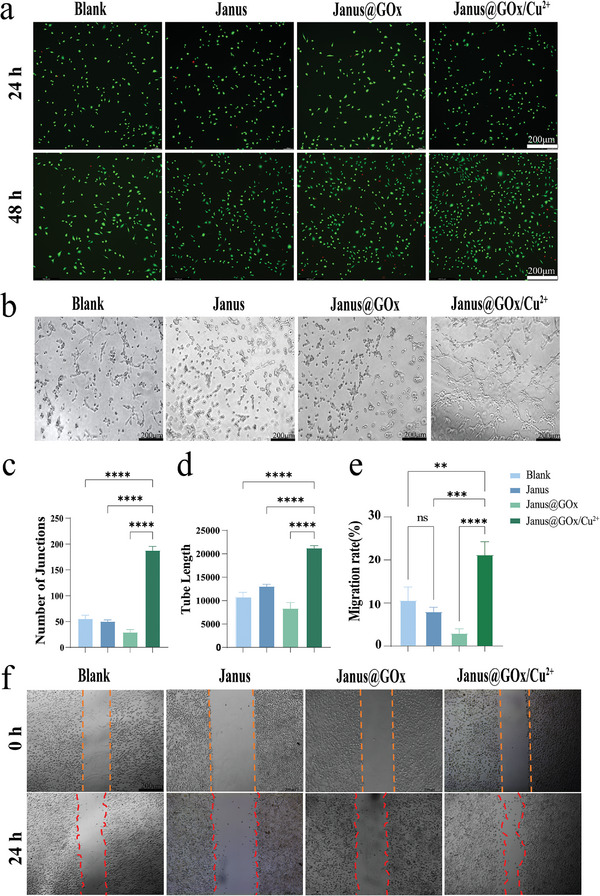
a) Fluorescent images of live‐dead staining of L929 cells after 24 and 48 h of coculture with membranes. b) Optical images of angiogenesis experiment. c,d) Quantification of the junctions and tube length. e) Quantification of migration rate in scratch experiment. f) Images of the scratch experiment at 0 h and 24 h. Scale bar, 200 µm. Data were expressed as mean ± SD. *n* ≥ 3, *p < 0.05; **p < 0.01; ***p < 0.001, ****p < 0.0001, and ns for no significant differences.

First, the cytocompatibility of the nanofiber membranes was verified using CCK‐8 and live‐dead staining. Fibroblasts L929 cells were coincubated with Janus and Janus@GOx membranes. L929 cells that were not co‐incubated with the materials were used as the blank group. As shown in Figure S10 (Supporting Information), the relative cell activities of all groups were above 80% at 24 and 48 h. As shown in **Figure** **5**a, the results of live/dead cell staining at 24 and 48 h were consistent with those of CCK‐8, indicating that Janus@GOx/Cu^2+^ was not significantly toxic and could be used as a wound dressing.

Microvascular disease is a common complication of diabetes. Reduced local neovascularization significantly delays the healing of diabetic wounds. Activation of angiogenesis is crucial for granulation tissue formation. Thus, the correction of impaired local angiogenesis is a key step in treating chronic diabetic wounds. Appropriate concentration of Cu^2+^ prompts the expression of vascular endothelial growth factor (VEGF) and hypoxia‐inducible factor‐1α (HIF‐1α), as well as the production of keratin and collagen. Therefore, it is possible to promote angiogenesis and stimulate the migration of keratinocytes and fibroblasts by introducing Cu^2+^ into diabetic wound dressings. In the present study, Cu^2+^ was added to a Janus@GOx/Cu^2+^ membrane, and its pro‐angiogenic effect was tested. After coculturing with the sample extract medium for 4 h, human umbilical vein endothelial cells (HUVECs) of the Janus@GOx/Cu^2+^ group formed the most significant number of tube junctions compared to the other three groups (Figure 5b). The number of tube junctions (Figure 5c) and tube length (Figure 5d) were also quantified. These results indicated that the Janus@GOx/Cu^2+^ group exhibited the most potent vasculogenic effect. The lower number of junctions in the Janus@GOx group compared to the blank and Janus groups could be attributed to the decreased endothelial cell activity and angiogenic capacity due to the unconsumed H_2_O_2_ in the Janus@GOx extract. The experimental results demonstrated that loading Cu^2+^ onto the Janus@GOx membranes significantly enhanced angiogenesis. A scratch experiment was performed using L929 cells to assess the effect of the membranes on cell migration. As shown in Figure 5f, Janus@GOx/Cu^2+^ exhibited a difference compared to the other control groups, with cells migrating towards the blank area and the area of the scratches decreasing. A quantitative analysis of the scratch experiment (Figure 5e) revealed a notably higher cell migration rate in the Janus@GOx/Cu^2+^ group than in the other groups. These results proved that Janus@GOx/Cu^2+^ played a role in promoting cell migration.

### 2.6 In Vivo Diabetic Infected Wound Healing Assessment

**Figure 6:**
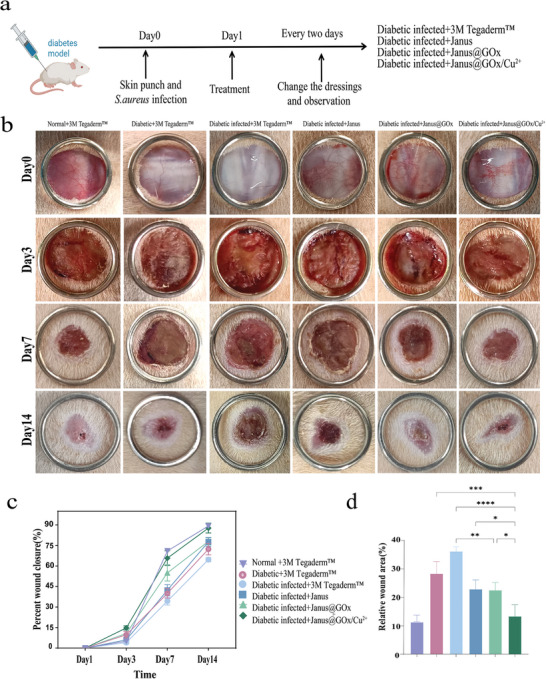
a) The protocol for creating and treating the diabetic infected wounds. b) Representative images of the diabetic wounds at different times. c) Quantification of wound contraction during the healing process. d) Quantification of relative wound area on day 14 after treatment. Data were expressed as mean ± SD. *n* ≥ 3, *p < 0.05; **p < 0.01; ***p < 0.001, ****p < 0.0001, and ns for no significant differences.

To assess the effects of the membrane material on wound disinfection and healing, diabetes‐infected wound models were created by adding *S. aureus* to the skin wound of diabetic rats (**Figure** **6**a). Additionally, uninfected wounds in healthy rats and diabetic rats were created as control groups, which were named the normal group and diabetic groups, respectively. 3 M Tegaderm Clear Wound Dressings provide excellent waterproof breathability and long‐lasting antimicrobial action for the care of all types of wounds, including superficial acute wounds and localized chronic wounds. Its effectiveness is recognized by the US Food and Drug Administration (FDA) and the Infusion Nurses Society (INS). Therefore 3 M Tegaderm Clear Wound Dressings were used as a control material to cover the wounds of the normal group, the diabetic group, and a group of diabetic infected to isolate and protect the wounds. The other three groups were covered with Janus, Janus@GOx, or Janus@GOx/Cu^2+^. As shown in Figure S11 (Supporting Information), numerous colony formations can be seen in the bacterial swab coating plate test of infected wounds, which demonstrate the successful construction of infection wound models. After treatment with different dressings, at Day 5, the number of colonies decreased in the Janus@GOx group and Janus@GOx/Cu^2+^ group compared to the control groups, with a more significant decrease in the JanusGOx/Cu^2+^ group, proving its excellent bactericidal effect. The trauma area and the dynamic healing process of the wounds on the 3^rd^, 7^th^, and 14^th^ day for each group are schematically shown in Figure 6b. The statistics of wound closure rate (Figure 6c) showed that the wound contraction on day 7 of the two groups treated with Janus@GOx and Janus@GOx/Cu^2+^ reached above 50%, which was far greater than that of the 3 M Tegaderm group. The Janus@GOx/Cu^2+^ group showed a wound closure rate of 65.75%, which was closed to that in the normal group (71%). On the 14^th^ day, the wound closure rate of the Janus@GOx/Cu^2+^ group (87.75%) was higher than that of the 3 M Tegaderm group (64.67%), approaching that of the normal group (89.25%) (Figure 6d). These results suggest that the Janus@GOx/Cu^2+^ membrane significantly promotes wound healing in diabetic infections.

### 2.7 Histological Analysis

The healing effect was also assessed histologically by hematoxylin and eosin (H&E) and Masson staining. On the 7^th^ day, the H&E‐stained sections revealed an inflammatory response in all groups, with inflammatory cells observed at the edge of the wound. After treatment with the Janus@GOx/Cu^2+^ membrane, the trauma showed excellent local reepithelialization, as evidenced by the reconstruction of the wound surface to form an entirely new epithelial structure. In the wounds of the Janus@GOx/Cu^2+^ group, a well‐organized arrangement of the stratum basale, spinosum, granulosum, lucidum, and corneum was observed, with a thick layer of keratin formed on the surface (Figure S12, Supporting Information). In contrast, Masson staining showed more extensive subepithelial deposition of collagen fibers in the Janus@GOx/Cu^2+^ group (Figure S13, Supporting Information). On day 14, the wounds in the Janus@GOx/Cu^2+^ group healed well. H&E and Masson staining showed that the thickness of the repaired epidermis on the wounds in the Janus@GOx/Cu^2+^ group was approximately the same as that of the normal skin, and the collagen fibers were arranged in a compact and orderly manner. The re‐epithelialization was also better in the Janus and Janus@GOx groups compared to the 3 M Tegaderm group. These results suggest that the epidermal formation in wounds treated with the Janus@GOx/Cu^2+^ membrane was significantly enhanced, indicating its role in promoting wound healing.

**Figure 7:**
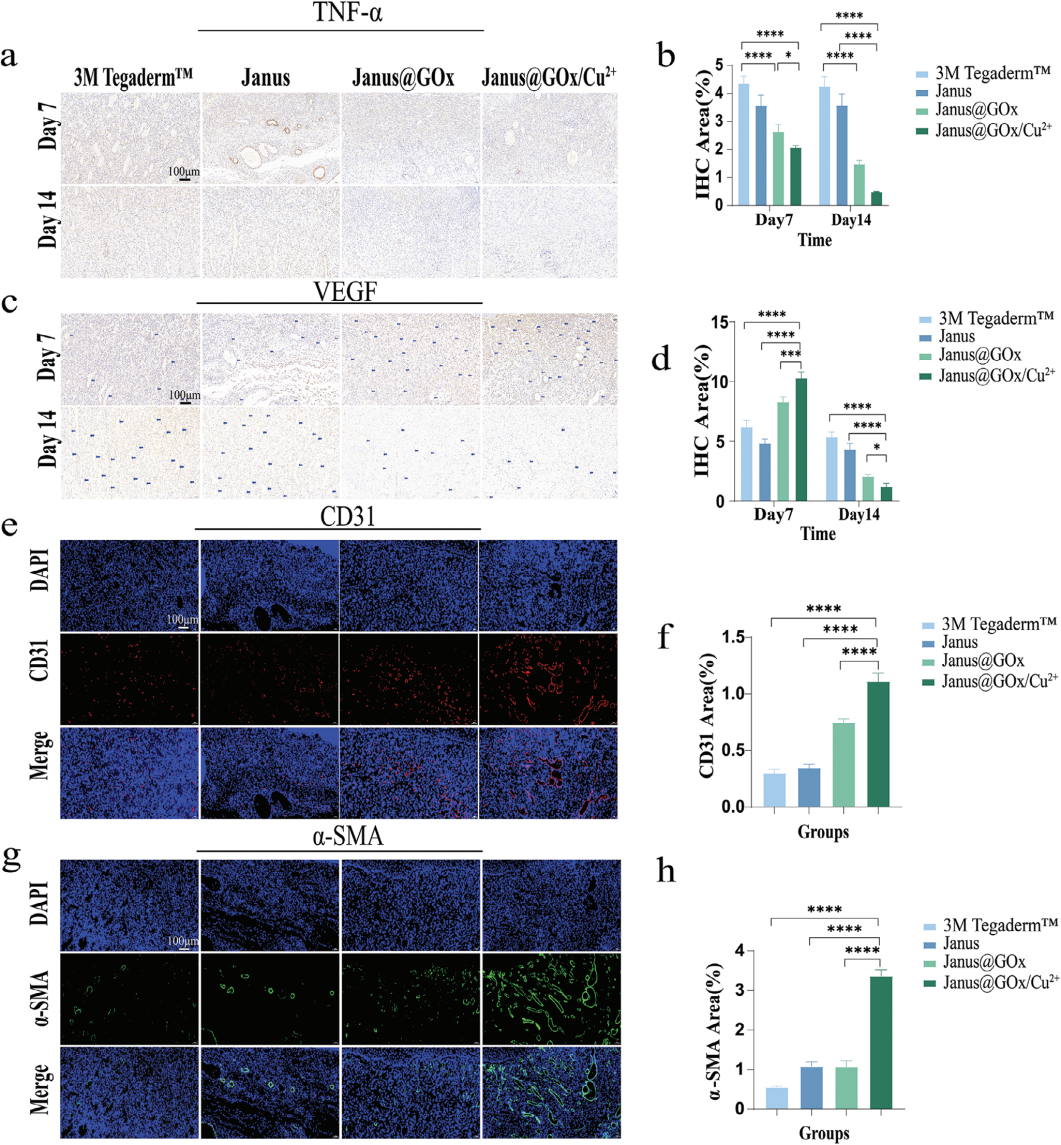
Histological analysis of wounds. a) TNF‐α expression in wounds on day 7 and 14, as determined by immunohistochemical staining. b) Quantification of the expression of immunohistochemical TNF‐α. c) VEGF expression in wounds on days 7 and 14, as determined by immunohistochemical staining. d) Quantification of the expression of immunohistochemical VEGF. e) CD31 expression in wounds on day 7, as determined by immunofluorescent staining. f) Quantification of the expression of immunofluorescent CD31. g) α‐SMA expression in wounds on day 7, as determined by immunofluorescent staining. h) Quantification of the expression of immunofluorescent α‐SMA. Scale bar, 100 µm. Data were expressed as mean ± SD. *n* ≥ 3, *p < 0.05; **p < 0.01; ***p < 0.001, ****p < 0.0001, and ns for no significant differences.

Healing in diabetic wounds is severely affected by persistent inflammation. The inflammatory factors cause the inflammatory cascade response that results in massive infiltration of inflammatory cells on the wound surface, delaying wound healing.^[^ ^24^ ^]^ The typical pro‐inflammatory cytokine tumor necrosis factor‐α (TNF‐α) is chosen to indicate the level of inflammation in the wound.^[^ ^25^ ^]^ TNF‐α can be stained brown by immunohistochemical staining. As shown in **Figure** **7**a,b, the expression of TNF‐α was lower in the Janus@GOx/Cu^2+^ group than in the other groups at days 7 and 14. Additionally, the expression levels of TNF‐α in the Janus@GOx group and Janus@GOx/Cu^2+^ group were significantly lower at day 14 compared to day 7. These results indicated that Janus@GOx/Cu^2+^ effectively reduced the expression of inflammatory factors in vivo, which had a significant positive effect on wound repair.

Neovascularization is an essential part of wound repair because blood vessels provide nutrients for the growth of granulation tissue.^[^ ^26^ ^]^ VEGF is an endothelial cell marker for capillary formation.^[^ ^25^, ^27^ ^]^ The expression level of VEGF was assessed by immunohistochemical staining to verify the role of Janus@GOx/Cu^2+^ in promoting angiogenesis. As shown in Figure 7c,d, the expressions of VEGF in the tissues of the Janus@GOx group and Janus@GOx/Cu^2+^ group were much higher than those of the 3 M Tegaderm group and Janus group on day 7, suggesting that Janus@GOx and Janus@GOx/Cu^2+^ had a significant promoting effect on angiogenesis during the proliferative phase. In contrast to the results obtained on day 7, the VEGF expression in the Janus@GOx and Janus@GOx/Cu^2+^ groups decreased on day 14. This was attributed to the completion of wound healing and neovascularization in both groups. The in vivo proangiogenic effect of Janus@GOx/Cu^2+^ was further verified by immunofluorescence labeling of the angiogenic marker CD31 and the vascular smooth muscle marker α‐smooth muscle actin (α‐SMA).^[^ ^28^ ^]^ As shown in the fluorescence images of CD31 (Figure 7e) and α‐SMA (Figure 7g) on day 7, the Janus@GOx/Cu^2+^ group exhibited better neovascularization. In contrast, the 3 M Tegaderm group and the Janus group exhibited only a few neovessels. Additionally, quantitative analysis of CD31 and α‐SMA fluorescence images (Figure 7f,h) showed that the average fluorescence intensity of the Janus@GOx/Cu^2+^ group was significantly higher than that of the other groups. The above results suggested that the Janus@GOx/Cu^2+^ membrane had a significant angiogenesis‐promoting effect.

## 3 Conclusion and Discussion

Diabetic ulcers are common and challenging complications in patients with diabetes. Conventional treatments have limitations; therefore finding effective strategies to promote diabetic wound healing is crucial. In this study, electrostatic spinning was used to prepare a three‐layered Janus membrane loaded with GOx and Cu^2+^. The Janus@GOx/Cu^2+^ membrane system combines a unidirectional pumping membrane with a glucose‐catalyzed degradation cascade reaction to impart multiple properties to wound dressings. These properties include the simultaneous reduction of exudates, local lowering of glucose, sterilization, and promotion of angiogenesis, making it suitable for managing infected diabetic wounds. The results of in vitro and in vivo experiments showed that Janus@GOx/Cu^2+^ significantly promoted diabetic wound healing.

Janus membranes with bifacial asymmetries have been rapidly developed in recent years and have tremendous potential for bioengineering and environmental applications. Asymmetric fabrication or modification imparts different properties to the bifacial surfaces, thereby enabling them to perform multiple functions.^[^ ^29^ ^]^ Many studies have reported that Janus membranes with asymmetric wettability can be used to manipulate the wetting and transport behavior of fluids to achieve unidirectional fluid transport. This allows the fluid to flow in one direction while preventing penetration in the opposite direction, a phenomenon known as the “fluid diode effect.”^[^ ^30^ ^]^ Based on this effect, Janus membranes have been widely used in the field of functional fabrics to manage humidity in the human skin.^[^ ^11^, ^12^, ^31^ ^]^ In the biomedical field, Janus membranes with targeted fluid delivery are also used as wound‐healing dressings to pump fluids out of wounds to reduce pain and healing difficulties caused by wound maceration. Additionally, they impart multiple prohealing properties to Janus membranes through the addition of bioactive factors.^[^ ^4^, ^10^, ^11^, ^32^ ^]^ There are two main types of Janus membranes: 1) monolayer Janus membranes containing two surfaces with opposite wettabilities, exhibiting a wettability gradient along the thickness of the material. 2) multilayer Janus membranes are obtained by fabricating each layer of the membrane separately and then combining them.^[^ ^29^ ^]^ Janus membranes are prepared by electrostatic spinning, gelation, and in situ modification. Electrostatically spun nanofiber materials are preferred because of their high specific surface area, high porosity, excellent hygroscopicity, and superior oxygen exchange capacity.^[^ ^33^ ^]^ By adjusting the composition of the spinning solution and spinning parameters, researchers have obtained electrospun nanofiber membranes that mimic the extracellular matrix structure of the skin, thus accelerating the adhesion and proliferation of epidermal cells.^[^ ^11^, ^33^, ^34^ ^]^ Electrostatically spun fiber membranes loaded with drugs, bioactive factors, and stem cells play an essential role in local drug delivery. They help avoid the curtailment of therapeutic effects caused by poor blood perfusion and other body circulation factors, meeting the multiple needs of wound healing.^[^ ^35^ ^]^ Researchers have designed three‐layer composite fabrics with water‐directional transport properties based on a gradient‐wetting structure using electrostatic spinning. These fabrics consist of hydrophobic, transition, and superhydrophilic layers.^[^ ^12^, ^36^ ^]^ The innovative introduction of a transition layer to build a multilayer Janus membrane guided the continuous transport of water. Water droplets are subjected to several forces during the transfer process, and the imbalance between the hydrophobic force and hydrostatic pressure causes the water to break through the hydrophobic side of the membrane.^[^ ^12b^ ^]^ The difference in capillary forces resulting from the significant wettability difference between the transition and superhydrophilic layers enables directional transport and diffusion of water, preventing the rewetting of the inner layer. In this study, three‐layer Janus membranes with gradient wettabilities were fabricated using electrostatic spinning. These membranes induced the migration and proliferation of fibroblasts and epithelial cells while unidirectionally pumping out excessive wound exudate, thereby promoting wound healing.

It has been shown that multiple factors contributing to difficult‐to‐heal diabetic injuries are reversible when blood glucose levels are controlled. Achieving exquisite blood glucose concentration control by performing continuous subcutaneous insulin injections can reduce hemoglobin glycosylation, inhibit collagen damage, and effectively improve wound healing.^[^ ^37^ ^]^ Wound dressings loaded with bioactive substances such as glucose oxidase, insulin, and photocatalysts are emerging. These dressings reduce glucose concentration in wounds through local decomposition or systemic regulation to alleviate the angiogenesis obstacle caused by hyperglycemia and the colonization of bacterial biofilms, while generating products such as ethanol and H_2_O_2_ to play a role in sterilization. They have significant therapeutic effects on the treatment of diabetes‐infected wounds.^[^ ^38^ ^]^ Localized excess glucose in diabetic wounds was decomposed by grafting glucose oxidase onto electrostatically spun fibers. This approach minimizes tissue damage and inhibits biofilm formation induced by high glucose concentrations.

Copper ions were introduced into the Janus membrane using a blending method. These ions underwent a Fenton‐like reaction with H_2_O_2_ produced by glucose decomposition and catalyzed the decomposition of H_2_O_2_ to release ·OH. The Fenton reaction is a redox reaction between H_2_O_2_ and Fe^2+^ that produces highly reactive ·OH. The product, ·OH, with much higher reactivity and oxidative power than H_2_O_2_ and singlet oxygen, plays an important role in killing both Gram‐negative and Gram‐positive bacteria. As alternatives to iron ions, copper‐based materials have been shown to exhibit Fenton‐like catalytic activity over a wide pH range of 3–7. Thus, Cu^2+^ was introduced into a diabetic wound dressing system due to its anti‐infective effects. The addition of a small amount of Cu^2+^ stimulates blood vessel formation and the migration of fibroblasts, promoting wound healing.

Overall, the three‐layer electrostatically spun Janus membrane demonstrated favorable performance in promoting diabetic wound healing. Its unidirectional permeability, antimicrobial properties, and proangiogenic effects were experimentally verified. In vivo experiments confirmed that the application of Janus@GOx/Cu^2+^ membranes to diabetic‐infected wounds significantly promoted wound healing, with a 14 d healing progression similar to that of normal mouse wounds and wound contraction rates approaching 90%. The findings of this study provide new directions and methods for wound treatment in patients with diabetes. However, despite the progress made in this study, more empirical studies on the feasibility of the clinical application of this material are required. Future optimization and improvement in the structure and properties of Janus membranes are believed to provide a more effective, safe, and feasible strategy for the treatment of diabetic wounds.

## 4 Experimental Section

### Materials

Polyacrylonitrile (PAN, *M*_w_ = 150000 g mol^−1^) and polyurethane (PU, *M*_w_ = 90000 g mol^−1^) were produced in Suzhou Cdh Achieves Fluorine Plastic Co., Ltd. N,N‐Dimethylformamide (DMF, 99.8%), glucose oxidase (GOx, ≥ 50 U mg^−1^ Lyophilized Powder), glutaraldehyde (GA, 50% in water), methyl alcohol (MT, 99.98%), N,N’‐dimethylthiourea (DMTU, >97%), hydrogen peroxide solution (H2O2, 30 wt% in H2O). All chemical reagents and solvents were reagent grade and used without further purification. Reverse osmosis (RO) water was used throughout all the experiments.

### Preparation of Modified Janus Membrane and Immobilization of Glucose Oxidase

The Janus membrane is constituted by three layers including hydrophilic layer, transfer layer, and hydrophobic layer, which is prepared by electrospinning layer by layer.

### Preparation of Spinning Solution

18 wt% PAN was added in DMF and stirred overnight to completely dissolve them as the spinning solution of the hydrophilic layer. Meanwhile 28 wt% PU particle was dissolved in DMF by stirring overnight and used as the component with hydrophobic properties. Besides, the PU solution with 5 mmol L^−1^ copper sulfate pentahydrate was prepared as the hydrophobic layer of the optimized material. The PAN solution and the pure PU solution were mixed in a ratio of 1:1 to obtain the spinning solution for the transfer layer.

### Preparation of Basic Nanofibrous Scaffolds

The prepared PAN solution and the mixed solution were electrospun for 20 mL and 10 mL volume respectively to obtain a bilayer membrane structure. Both the solutions were spun with a 23 G blunt‐end needle at 17 kV high voltage and ‐2 kV negative voltage, and the feeding rate was 0.15 mL min^−1^. The distance between the needle tips and the collector was 20 cm, and the rotation rate of collector was 140 rpm. The temperature and humidity of the environment during electrospinning were 30 °C and 85%, respectively. The membrane was then immersed in 75% sodium hydroxide ethanol solution at 60 °C for 5 min. During this process, PAN was hydrolyzed by the high‐temperature alkaline condition resulting in a rougher fibrous surface structure and enhanced hydrophilic properties. The membrane was then removed from the solution, washed repeatedly in RO water, and dried in the oven at 60 °C.

### Grafting of Glucose Oxidase

The solution used for the reaction to take place consists of 80% methanol, 10% N, N’‐dimethylthiourea (0.5 mol L^−1^), 5% sodium hydroxide (1 mol L^−1^), and 5% solution of hydrogen peroxide (30%). The dried bilayers were first reacted in the solution without hydrogen peroxide for 5 min, hydrogen peroxide was then added and stored at room temperature for 3 h. The modified membranes were washed with methanol and distilled water and then submerged in a 25% glutaraldehyde solution for 1 h at 4 °C. Subsequently, the bilayers were repeatedly washed and placed in a 0.1% solution of glucose oxidase overnight at 4 °C. This solution was prepared by phosphate‐buffered saline (PBS) at pH 6. Finally, the membranes were removed, washed gently and repeatedly by the same buffer, and dried naturally in a ventilated area.

The trilayer membranes without loading of copper ions and glucose oxidase were named Janus group. The membrane grafted with glucose oxidase is named Janus@GOx group, and the membrane loaded with GOx and containing Cu^2+^ in the hydrophobic layer is named Janus@GOx/Cu^2+^ group.

### Fundamental Characterization of the Nanofiber Membranes

The chemical compositions of the membranes were characterized by Fourier transform infrared spectrometer (FTIR, Thermo Scientific Nicolet iS50, USA). The microstructure was observed by scanning electron microscope (SEM, JSM‐7500F, Japan). Thermogravimetric analysis (TGA) was carried out to analyze the thermal stability of polymeric membrane materials by a thermos gravimetric analyzer (TGA8000, PerkinElmer, USA).

### Characterization of the Membrane Water Transport Behavior

Liquid unidirectional permeability was characterized by water contact angle (WCA), water breakthrough pressure, and bilateral water wettability. The WCAs of membranes were measured by the optical tensimeter (Theta T200 device, Biolin Scientific, Sweden) at room temperature. The breakthrough pressure of membranes was obtained by measuring the maximum height of water column that the membranes could support. The breakthrough pressure of the membrane was measured by measuring the maximum height of the water column that the membrane can sustain. The diffusion and transfer of water were recorded from the side by slowly adding blue homogeneous dye solution drop by drop on both sides of the membrane of the same area.

### GOx Activity and Fenton‐Like an Activity Evaluation: Glucose Concentration Measurement

The activity of glucose oxidase was evaluated by the 3,5‐dinitrosalicylic acid (DNS) colorimetric method to demonstrate the successful grafting of enzyme on nanofiber membranes. The glucose solutions at a concentration of 2.5 g L^−1^ of pH 5.5 and pH 8.5 were prepared using analytical pure glucose, respectively. The reaction was carried out by adding the sample of 0.5 × 0.5 cm^2^ per mL of glucose solution as a ratio. The solution was shaken and reacted at a constant temperature of 37 °C and 0.5 mL of the solution was taken out at 6 h/12 h/24 h, respectively into a clean test tube. Subsequently, 0.5 mL of DNS reagent was added and mixed thoroughly. After heat in boiling water for 5 min, cooled rapidly with running water. Then mixed into 4 mL of deionized water and taken out 200 µL of the solution into 96‐well plate to detect the absorbance at 540 nm. The glucose standard curve was obtained by gradient dilution of 2.5 g L^−1^ glucose solution, and the linear regression equation of glucose concentration versus absorbance was also obtained by DNS colorimetric method to further calculate the glucose consumption at each time point.

### Detection of Hydrogen Peroxide Concentration

The concentration of hydrogen peroxide was measured by the colorimetric method of titanium sulfate. The titanium sulfate solution turns yellow after reacting with hydrogen peroxide, and its absorbance has a linear relationship with the concentration of hydrogen peroxide. The assay solution was prepared by dissolving 1.33 mL Ti (SO_4_)_2_ solution with 24% concentration and 8.33 mL H_2_SO_4_ in 50 mL deionized water. 50 µL of the sample solution was mixed with 100 µL of the assay solution for 30 min and the absorbance was measured at 405 nm. The standard curve was obtained by gradient dilution of 30% analytically pure hydrogen peroxide solution, which was used to calculate the concentration of hydrogen peroxide in the sample solution.

### pH Measurement

The pH changes of the solution were detected by pH detector. The starting pH of the glucose solution was 5.5 and 8.5 to simulate the acute wound and chronic wound microenvironment, respectively. The pH changes were accurately measured by pH detector after 6 h/12 h/24 h reaction with the samples at a constant temperature of 37 °C.

### Detection of ^•^OH

The glucose solutions at a concentration of 2.5 g L^−1^ of pH 5.5 and pH 8.5 were prepared using analytical pure glucose, respectively. The reaction was carried out by adding the 12 µL TMB solution (10 µg mL^−1^ in DMSO) and samples of 0.5 × 0.5 cm^2^ and per mL of glucose solution as a ratio. The color of the solution was observed after 24 h simultaneously measuring the absorption spectrum at 500–800 nm and the absorbance value at 652 nm.

### Antibacterial Activity

*E. coli* and *S. aureus* were utilized to test the antibacterial properties of the Janus membrane systems. Four groups were used for control experiments: including a blank group, the Janus membrane group, the Janus@GOx group, and the Janus@GOx/Cu^2+^ group. The bacterial liquid medium consisted of beef meal, peptone, and sodium chloride, and 2.5 g L^−1^ of glucose was added to simulate the diabetic wound environment. The solid medium was prepared by adding agar into the liquid medium, fully dissolved and high temperature sterilized, then poured into Petri dishes and placed under aseptic conditions at room temperature for 2 d before use. Resuscitate the strain and dilute the *S. aureus* solution and *E. coli* solution with culture medium to 10^6^ and 10^5^ CFU mL^−1^, respectively. The samples were cut to 0.5 × 0.5 cm2 membranes and cocultured with 1 mL bacterial solution at a constant temperature of 37 °C. After 6 h and 12 h, 200 µL bacterial solution of each well was removed and placed in 96‐well plate, to measure the absorbance at 600 nm. Subsequently, colony counting experiment was conducted to further verify the bactericidal properties of the materials. Briefly, the bacterial solutions, which were cocultured with the samples, were diluted with 10^5^ times normal saline. 100 µL of the diluted solutions were evenly spread on agar medium, and the number of bacterial colonies was counted after 16 h of incubation at 37 °C. The fiber membranes of each group were cocultured with bacteria for 12 h and then immersed in glutaraldehyde for 12 h for fixation. Subsequently, gradient alcohol dehydration was performed, and after drying, the morphologies of bacteria on the fiber membranes were observed by SEM.

### Evaluation of Biocompatibility In Vitro

1 × 10^4^ mouse fibroblast L929 cells were inoculated in each well of 24‐well plates and cultured for 24 h to enable adherent growth. After sufficient sterilization by UV light irradiation for 1 h, 0.5 × 0.5 cm^2^ size membranes were taken in 24‐well plates and cocultured with adherent fibroblasts in 1 mL of medium with 2.5 g L^−1^ glucose content (prepared using DMEM high sugar medium and RMPI1640 medium), during which the medium was changed every 8 h, and after 24 h and 48 h, the medium and the material were removed. The Cell‐Counting Kit‐8 (CCK‐8) original solution was diluted with the cell medium at a volume ratio of 1:9 and 400 µL of the mixed solution was added to each well and incubated at 37 °C. After 90 min, 200 µL of each well was removed for absorbance detection at 450 nm. The relative cell growth rate was calculated by the following equation:

(1) $$\mathrm{Relative}\;\mathrm{growth}\;\mathrm{rate}\left(\%\right)=\left(A_{s}-A_{c}\right)/\left(A_{\mathrm{nc}}-A_{c}\right)\times 100\%$$

where *A*_s_, *A*_nc_, and *A*_c_ are absorbances of the sample, negative control, and CCK‐8 diluted solution, respectively.

The cell activity was also examined by live‐dead staining. The medium was sucked and 200 µL of calcein‐AM/propidium iodide dye was added into each well. After 15 min, cells were observed by a fluorescent microscope (Carl Zeiss Observer 7, Germany).

### Assessment of Angiogenesis

Tube formation test using human umbilical vein endothelial cells (HUVECs) was performed to study the proangiogenic effects. In detail, after sterilization, the membranes were cut as 0.5 × 0.5 cm^2^ and immersed in 1 mL RMPI 1640 basic medium and leaching for 48 h to prepare extracts. The 50 µL of Matrigel was added into the wells of a 48‐well followed by gel under 37 °C for 30 min. Then 3 × 10^4^ cells were starved overnight in serum‐free medium and 1 mL extract was added onto Matrigel and cocultured for 4 h. The state of angiogenesis is photographed with an inverted microscope. And the number of nodes and branches was calculated using ImageJ software.

### Assessment of Cell Migration

The scratch experiment was carried out using L929 cells to investigate the impact of cell migration. The L929 cells were inoculated at a density of 5 × 10^4^ cells per well in six‐well plates and cultured for 24 h until they were completely spread out on the plates. Three straight lines were carefully created longitudinally and horizontally in each well using the tip of a sharp pipette gun. These lines intersected to form nine intersections in total. The nonadherent cells were removed by gently washing the well plates once with culture solution. Each group of material extract was then separately added for coculture, and the process of cell migration was observed under a microscope. Images were captured at both the initial time point and after 24 h to document the migration of the cells.

### In Vivo Diabetic Wound Healing Assessment

All procedures involving the use of animals in this study were performed in strict accordance with the Guide for the Care and Use of Laboratory Animals. All animal experiments were prospectively approved by the Ethics Committee of West China Hospital of Stomatology, Sichuan University (WCHSIRB‐D‐2023‐345).

The prowound healing effect of membrane materials was evaluated in the model of diabetic SD rats. After 12 h of fasting, streptozotocin (55 mg kg^−1^) was injected intraperitoneally into 40 male SD rats to induce a diabetic model. After 2 weeks, the rats with blood glucose concentrations higher than 16.9 mmol L^−1^ were determined to be diabetic. After shaving the hair on the back, these rats were randomly divided into five groups and whole skin wounds with a diameter of 15 mm were made on their backs. Four groups of rats were injected with 50 µL of *Staphylococcus aureus* bacterial solution (1 × 10^8^ cell mL^−1^) on the wound and treated with 3 M Tegaderm Clear Wound Dressings, Janus membranes, Janus@GOx, Janus@GOx/Cu^2+^. The remaining group was used as a diabetic rat control group without *S. aureus* and the wound was treated with 3 M Tegaderm Clear Wound Dressings. In addition, a control group of healthy rats with a 15 mm size wound on the back treated with 3 M dressings was added. The wound fluid before and after the treatment was collected and cultured on agar plates to determine the antibacterial activity. The wound sites were observed and photographed on days 1, 3, 7, and 14 postwounding. Wound areas were measured and analyzed by Image J software. The wound contraction rate was calculated by the following equation:

(2) $$\mathrm{Wound}\;\mathrm{contraction}\;\left(\%\right)=\left(S_{0}-S_{n}\right)/S_{0}\times 100\%$$

where *S*_0_ and *S*_n_ represent the initial wound area and wound area at different time points, respectively.

### Histology and Immunohistochemistry

Rats were sacrificed and the regenerated skin samples were excised and collected on day 3, day 7, and day 14. Skin samples were embedded in paraffin after fixation and gradient dehydration. Subsequently, 5 µm sections were prepared for hematoxylin & eosin (H&E) and Masson staining. On the corresponding days, skin wound tissue was excised for immunohistochemical and immunofluorescence evaluation, respectively. To assess the effect of proangiogenesis, VEGF was detected by the immunohistochemical method, and CD31 and α‐SMA were detected by the immunofluorescence method. In addition, immunohistochemical staining for TNF‐α and HIF‐α was also used to detect changes in the local inflammatory microenvironment of wounds. The quantification of immunohistochemical was counted by ImageJ software.

### Statistical Analysis

All experiments were performed at least three independent experiments. The data were presented as the mean ± standard deviation. One‐way single factorial analysis of variance (ANOVA) was performed to determine the statistical significance of the data. The statistical significance of the differences was expressed as *P* values * < 0.05, ** < 0.01, *** < 0.001, **** < 0.0001, and ns for no significant differences.

## Conflict of Interest

The authors declare no conflict of interest.

## Author Contributions

The manuscript was written through the contributions of all authors. All authors have given approval to the final version of the manuscript. Y.L.: conceptualization, methodology, validation, formal analysis, investigation, data curation, writing—original draft, visualization. W.W.: methodology, investigation, writing—review & editing. K.Q.: methodology, investigation. Y.W.: methodology, investigation. W.Z.: conceptualization, resources, writing—review & editing, supervision, project administration. H.X.: resources, methodology, conceptualization, supervision, funding acquisition, C.Z.: supervision.

## Supporting information

Supporting Information

Supplemental Movie 1

Supplemental Movie 2

## Data Availability

Data sharing is not applicable to this article as no new data were created or analyzed in this study.
