# Supplementary material for: Exudate Unidirectional Pump to Promote Glucose Catabolism Triggering Fenton‐Like Reaction for Chronic Diabetic Wounds Therapy
Source: Adv Sci (Weinh). 2024 Aug 9;11(38):2404652. doi: 10.1002/advs.202404652 (PMC11481212; doi:10.1002/advs.202404652)
Supplement: Supplementary file 1 — Supporting Information [file ADVS-11-2404652-s003.docx]

Supporting Information

**Exudate Unidirectional Pump to Promote Glucose Catabolism Triggering Fenton-like Reaction for Chronic Diabetic Wounds Therapy**

Yaxian Liang^a^, Wenjie Wang^b^, Kailong Qi^a^, Yige Wei^a^, Weifeng Zhao^b^, Huixu Xie^a^,*, Changsheng Zhao^b^,*

*^a^ State Key Laboratory of Oral Diseases, National Clinical Research Center for Oral Diseases, West China College of Stomatology, Sichuan University, Chengdu 610041, China*

*^b^ College of Polymer Science and Engineering, State Key Laboratory of Polymer Materials Engineering, Sichuan University, Chengdu 610054, China.*


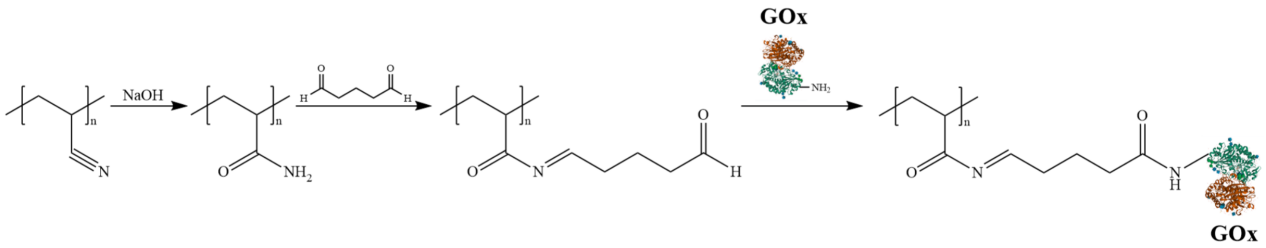


**Figure S1.** Chemical formula of the grafting reaction process


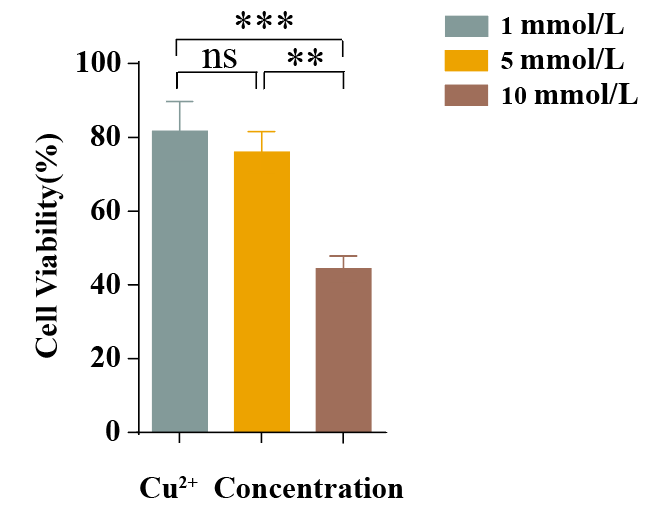


**Figure S2.** Cell viability after 24 hours of co-culture with Janus@GOx/Cu^2+^ membranes containing different Cu^2+^ concentrations.


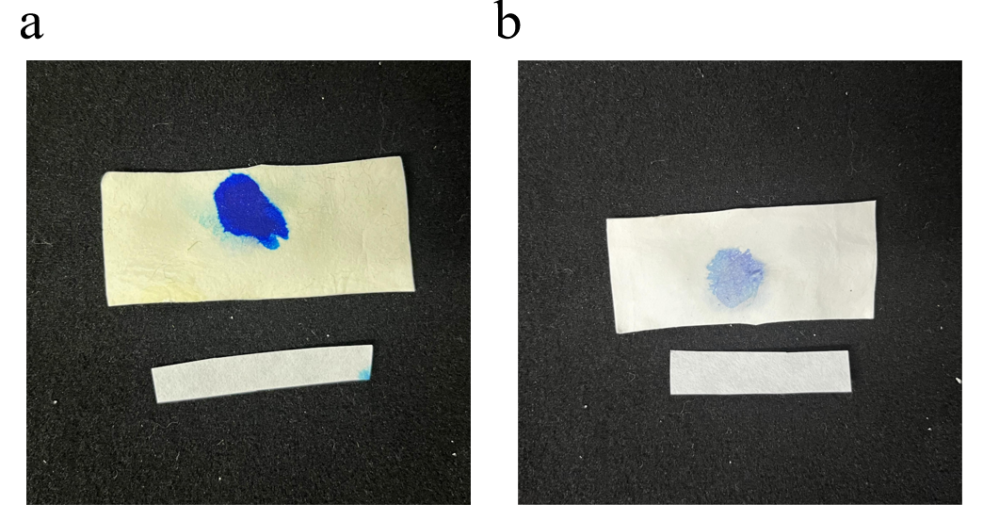


**Figure S3.** Degree of penetration of the superhydrophilic side (a) and hydrophobic side (b) of Janus@GOx/Cu^2+^ after ink penetration experiments.


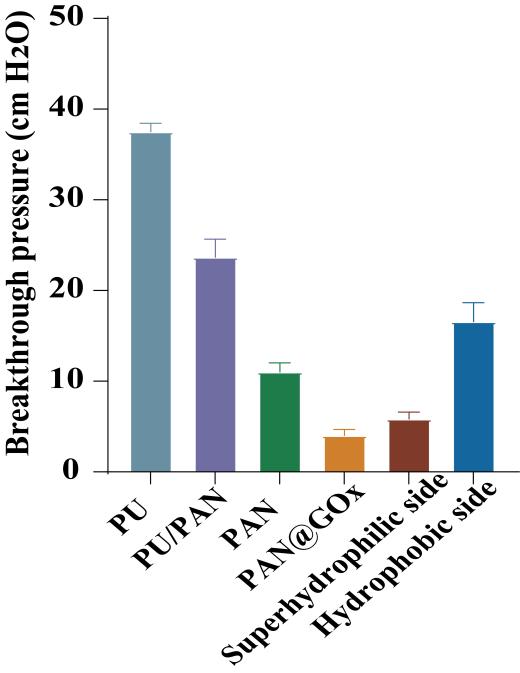


**Figure S4.** Water breakthrough pressure of different components of electrostatically spun fiber membranes as well as superhydrophilic side and hydrophobic side of Janus@GOx/Cu^2+^ membrane.


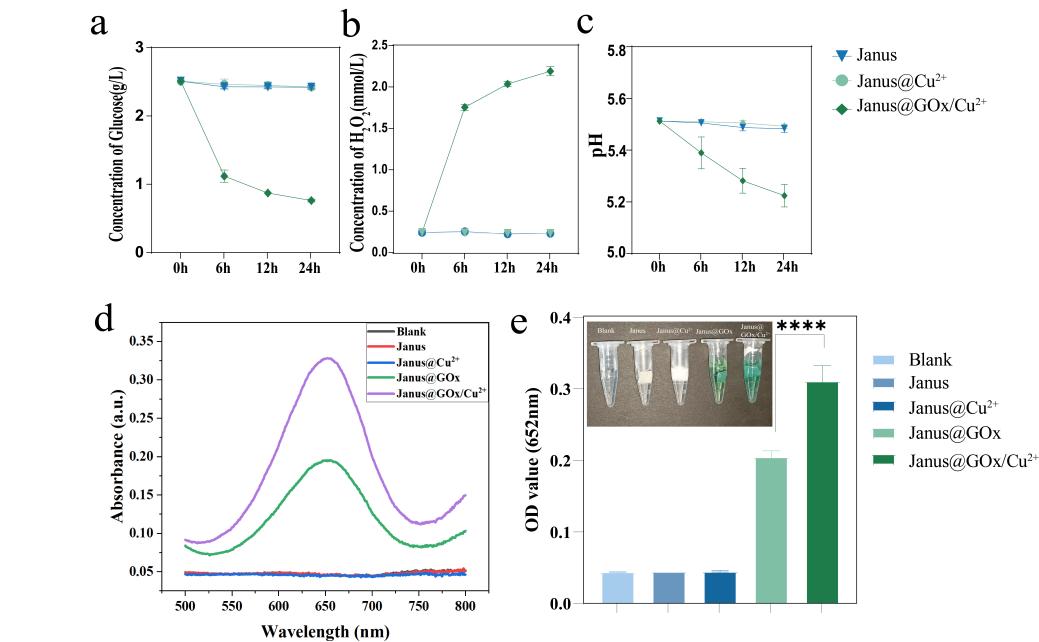


**Figure S5.** a-c) Changes of glucose concentration, H_2_O_2_ concentration and pH in pH=5.5 environment .d) Absorption spectra of TMB chromogenic reaction in pH=5.5 environment at 500-800 nm. e) Absorbance values of TMB chromogenic reaction in pH=5.5 environment at 652 nm.


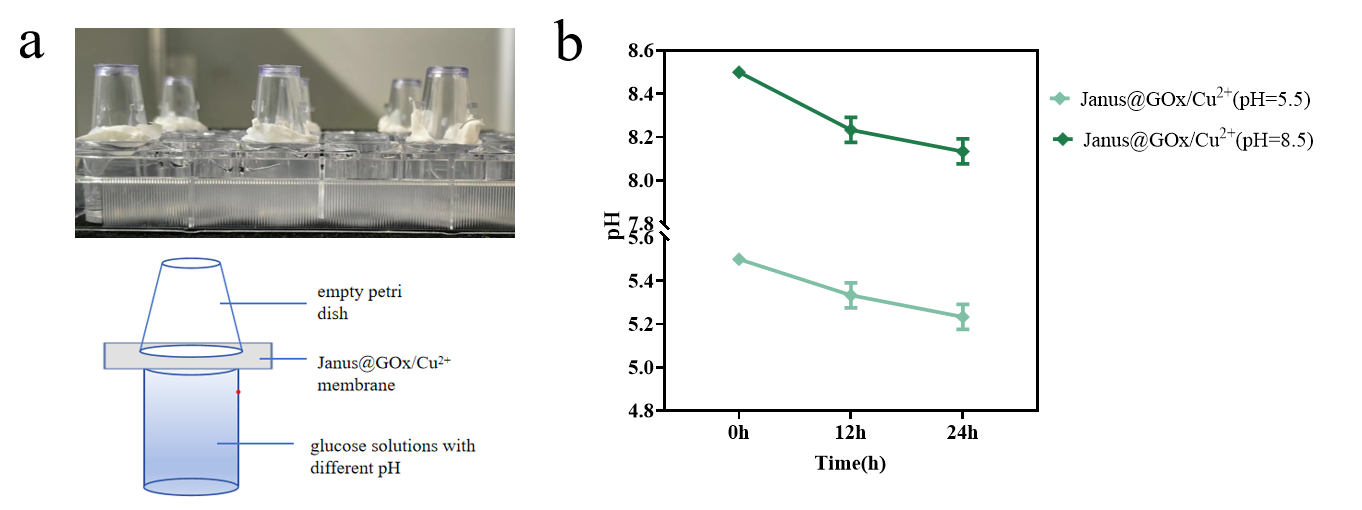


**Figure S6**. The experiment on pH regulation by diffusion of glucuronic acid. a) The device of diffusion experiment. b) The changes of pH after 12 and 24 h in the different acid-base environment.


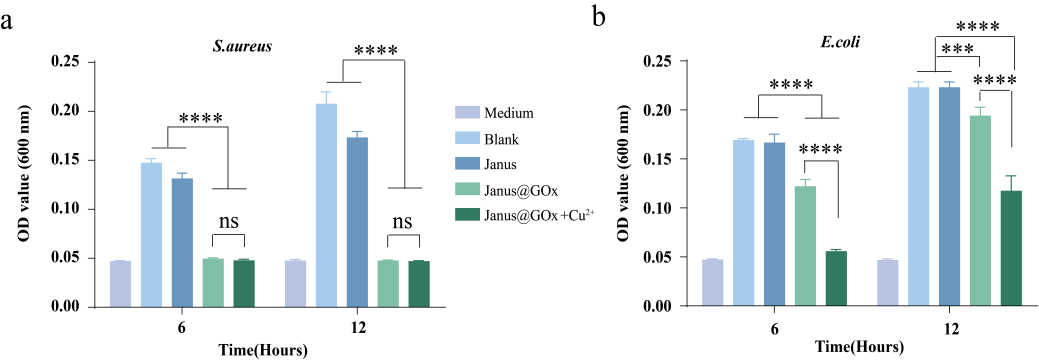


**Figure S7**. Absorbance values of *S. aureus* fluids(a) and *E. coli* fluids(b) after co-culture with nanofiber membranes.


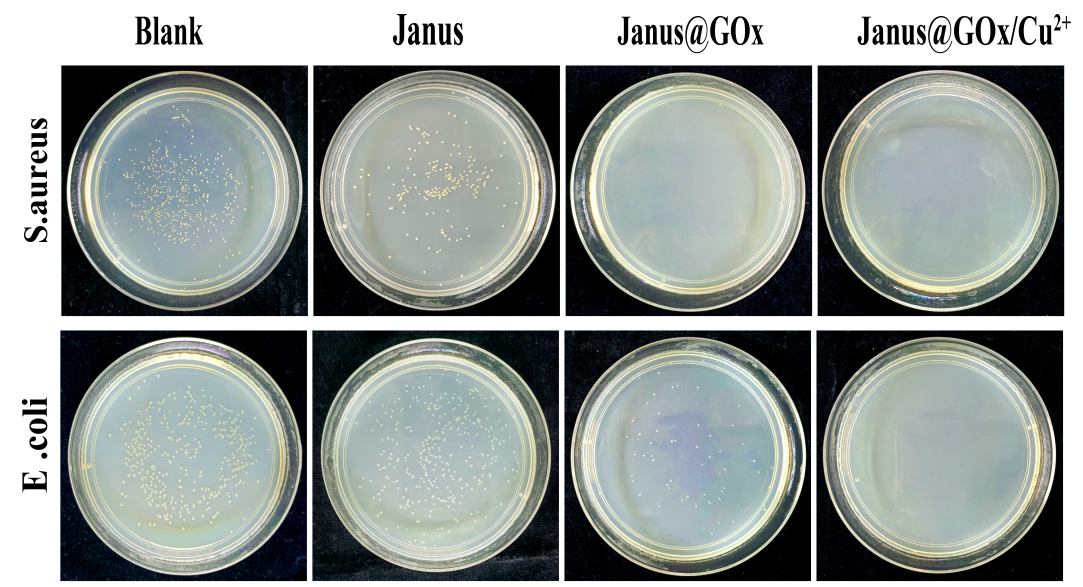
 **Figure S8.** Plate counting pictures of *S. aureus* and *E. coli* after co-culture with nanofiber membrane at 6h.


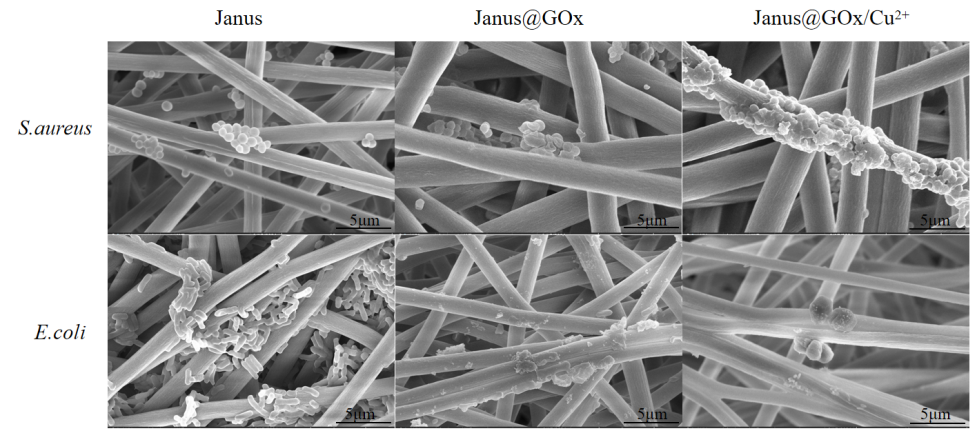


**Figure S9.** The morphology of *S.aureus and E.coli* were observed by SEM after 12 h of co-culture with the membranes. Scale bar, 5 µm.


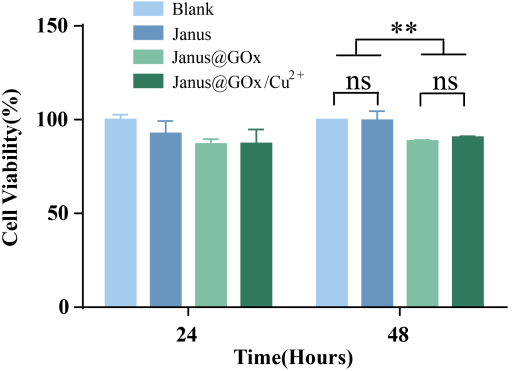


**Figure S10.** Cell viability after 24 hours and 48 hours of co-culture with different spinning membrane.


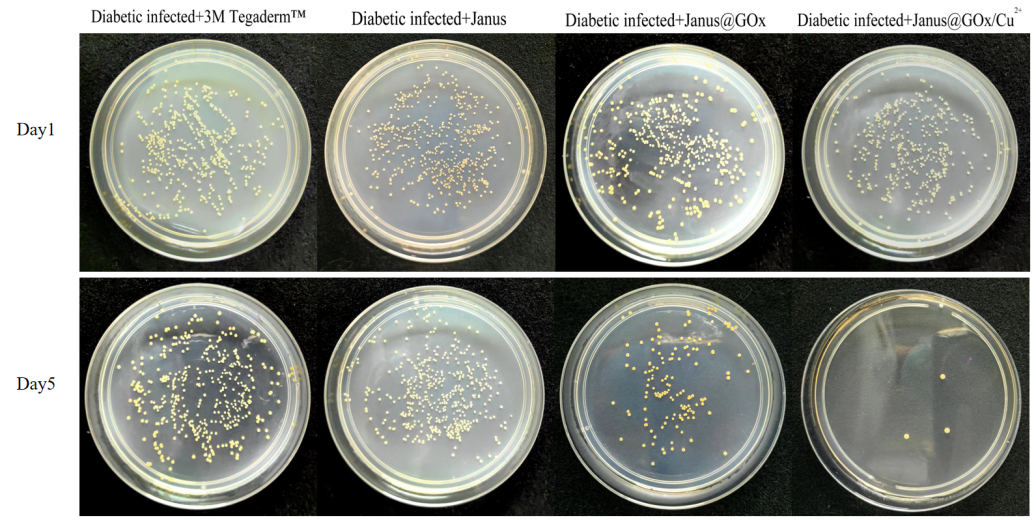


**Figure S11.** Images of live bacterial colonies in each group of diabetic infected wounds before (Day 1) and after (Day 5) dressings treatment.


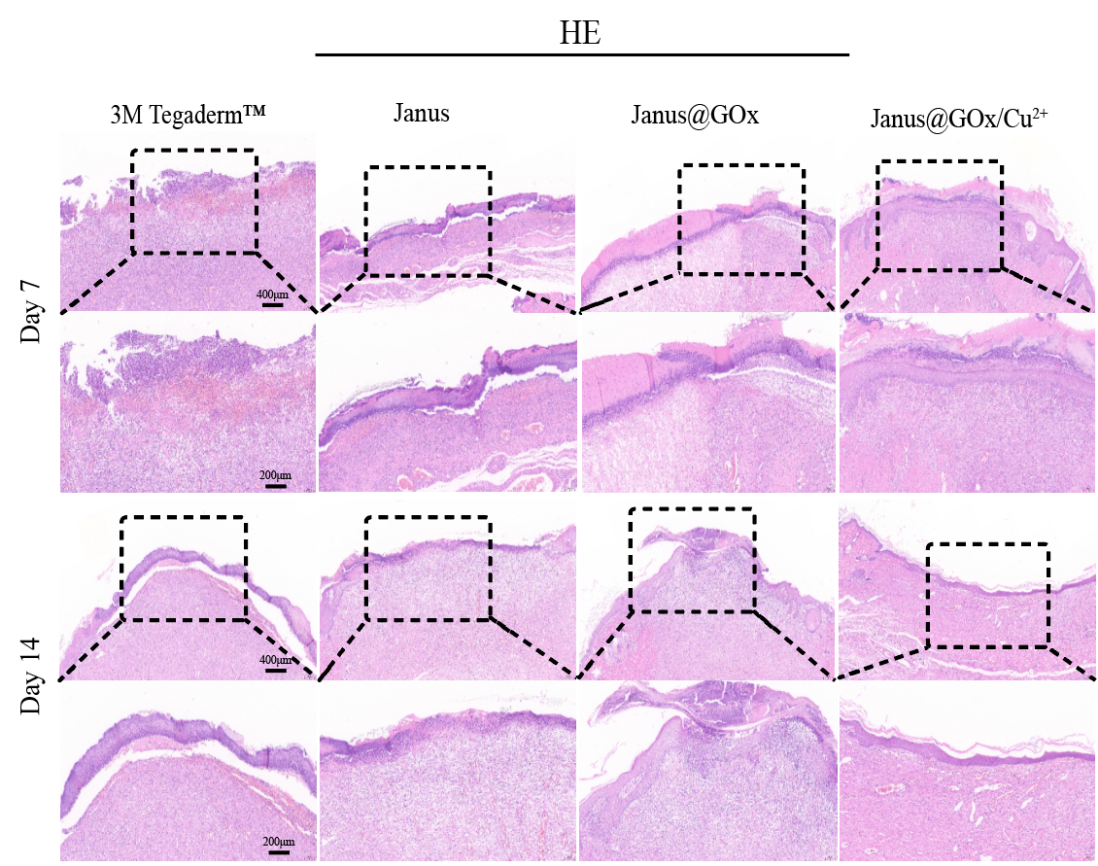


**Figure S12.** H&E staining of wound sections on day 7 and 14. Scale bars are marked in the pictures.


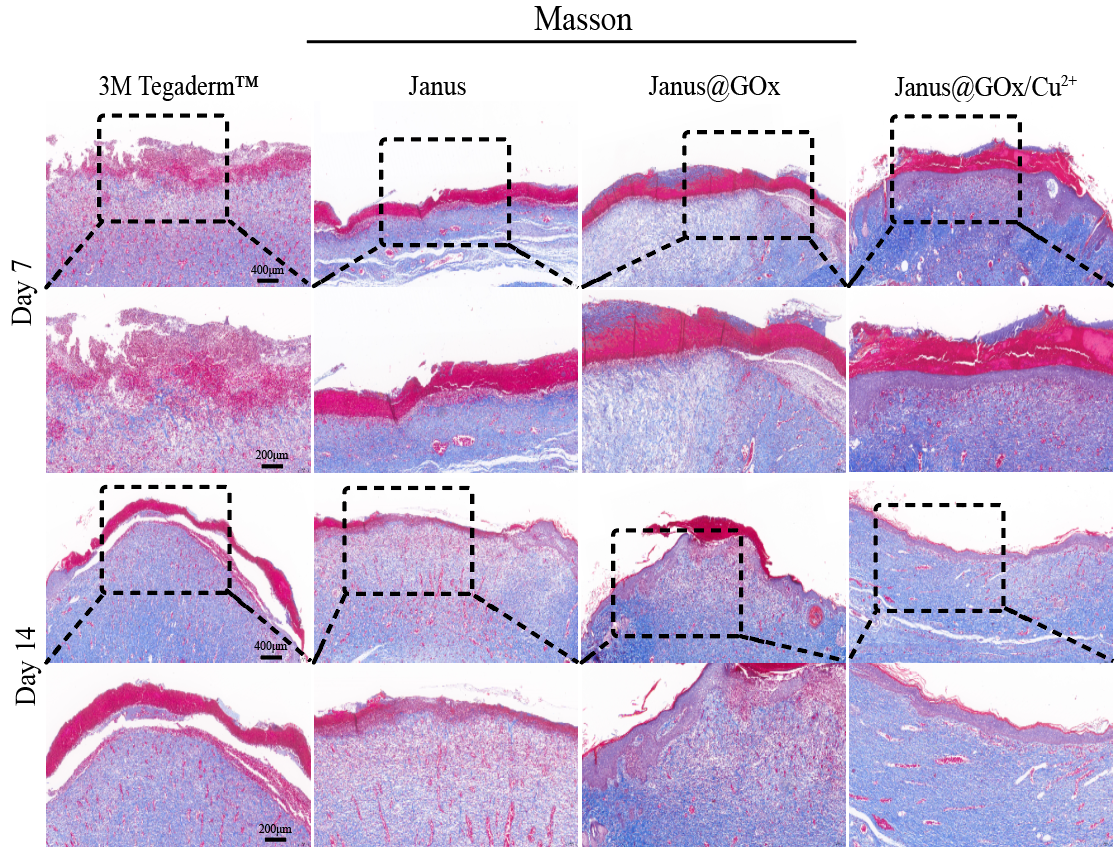


**Figure S13.** Masson staining of wound sections on day 7 and 14. Scale bars are marked in the pictures.

**Movie S1.** Ink reverse osmosis experiment of the superhydrophilic side.

**Movie S2.** Ink reverse osmosis experiment of the hydrophobic side.
